# Management of a Rhegmatogenous Retinal Detachment Involving the Macula in Senile Retinoschisis: A Case Report

**DOI:** 10.7759/cureus.105430

**Published:** 2026-03-18

**Authors:** Meshari I Alshabri, Basel M Alsolami, Mohammad I Almatrafi, Mariam M Tunkar, Nasser Al Saedi

**Affiliations:** 1 Ophthalmology, King Abdullah Medical City, Makkah, SAU; 2 Ophthalmology, Al-Noor Specialist Hospital, Makkah, SAU; 3 General Practice, Rabigh General Hospital, Rabigh, SAU

**Keywords:** combined retinoschisis-detachment, pars plana vitrectomy (ppv), peripheral retinoschisis, retinal detachment repair, retinectomy

## Abstract

In this report, we present a case of retinal detachment (RD) in the lower outer inferotemporal area involving the macula in the right eye, along with retinoschisis extending up to the inferior arcade in the left eye. A 59-year-old, medically fit female was referred to our center with a diagnosis of right-eye RD in the lower outer inferotemporal area involving the macula, along with retinoschisis extending up to the inferior arcade.

In this brief case report, we demonstrate successful management, with a favorable anatomical and functional outcome of a retinoschisis-related rhegmatogenous retinal detachment (RRD). The current case highlighted the presentation, anatomical outcomes, and functional outcomes with treatment modalities of retinoschisis-associated RD.

## Introduction

Senile retinoschisis is a peripheral retinal disorder affecting 1.4%-4% of individuals over 40 years of age [1,2]. It is characterized by a splitting between the inner and outer layers of the retina, mainly the neurosensory retina in the outer plexiform layer, and it is associated with cystic degeneration [1,3]. In most patients, retinoschisis remains asymptomatic, but in 0.5%, progressive symptomatic retinal detachment (RD) arises [4]. Regarding all RDs, retinoschisis accounts for only 1.4% [5]. In retinoschisis, the RD is usually confined to the zone of the retinoschisis. However, in less than 1% of patients, RD progresses beyond the borders. Rhegmatogenous retinal detachments (RRDs) associated with retinoschisis occur due to the presence of a full-thickness retinal tear or outer and inner breaks that allow the liquefied vitreous to pass under the retina.

## Case presentation

**Figure 1:**
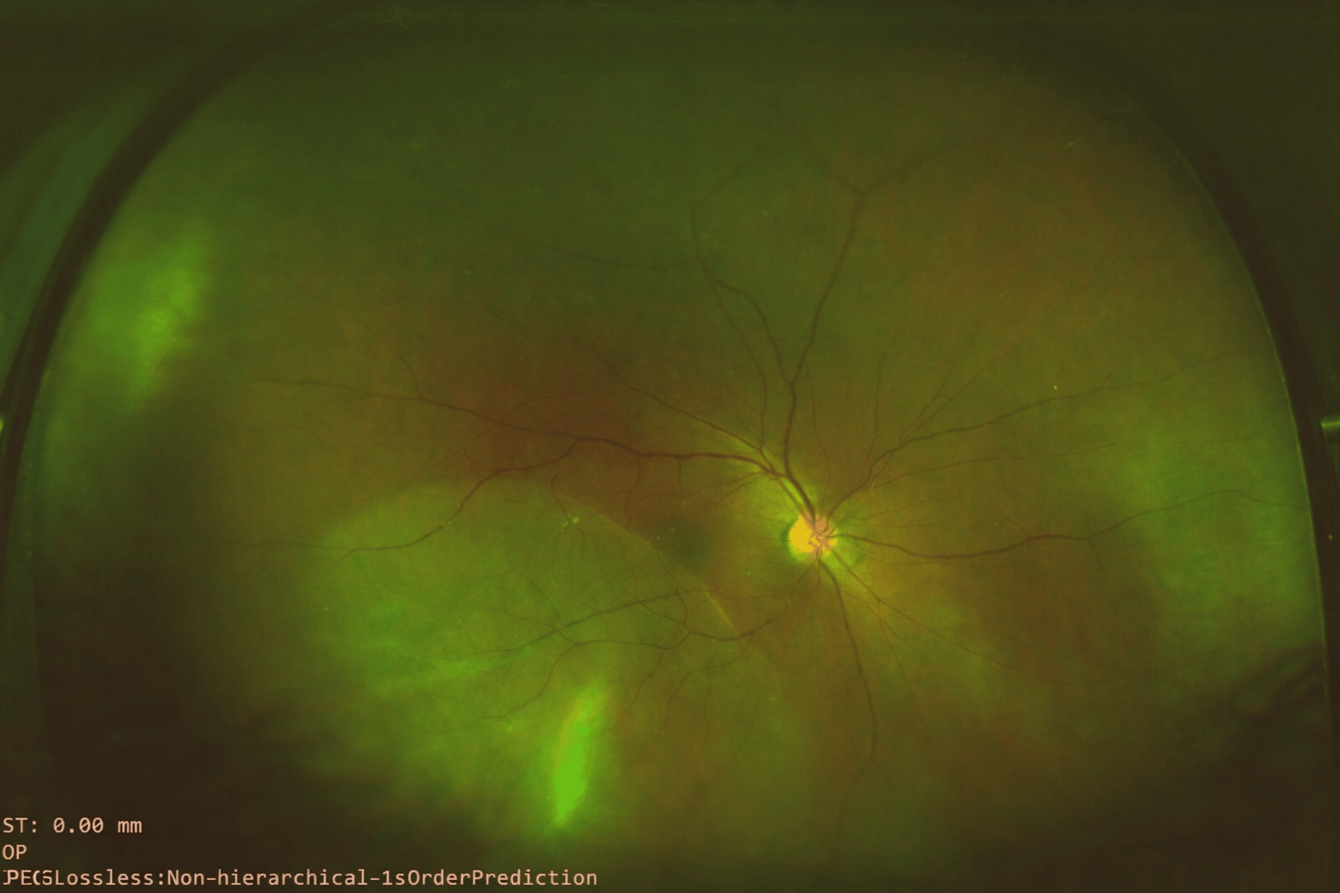
A preoperative wide-field fundus photo showing rhegmatogenous retinal detachment involving the macula.

**Figure 2:**
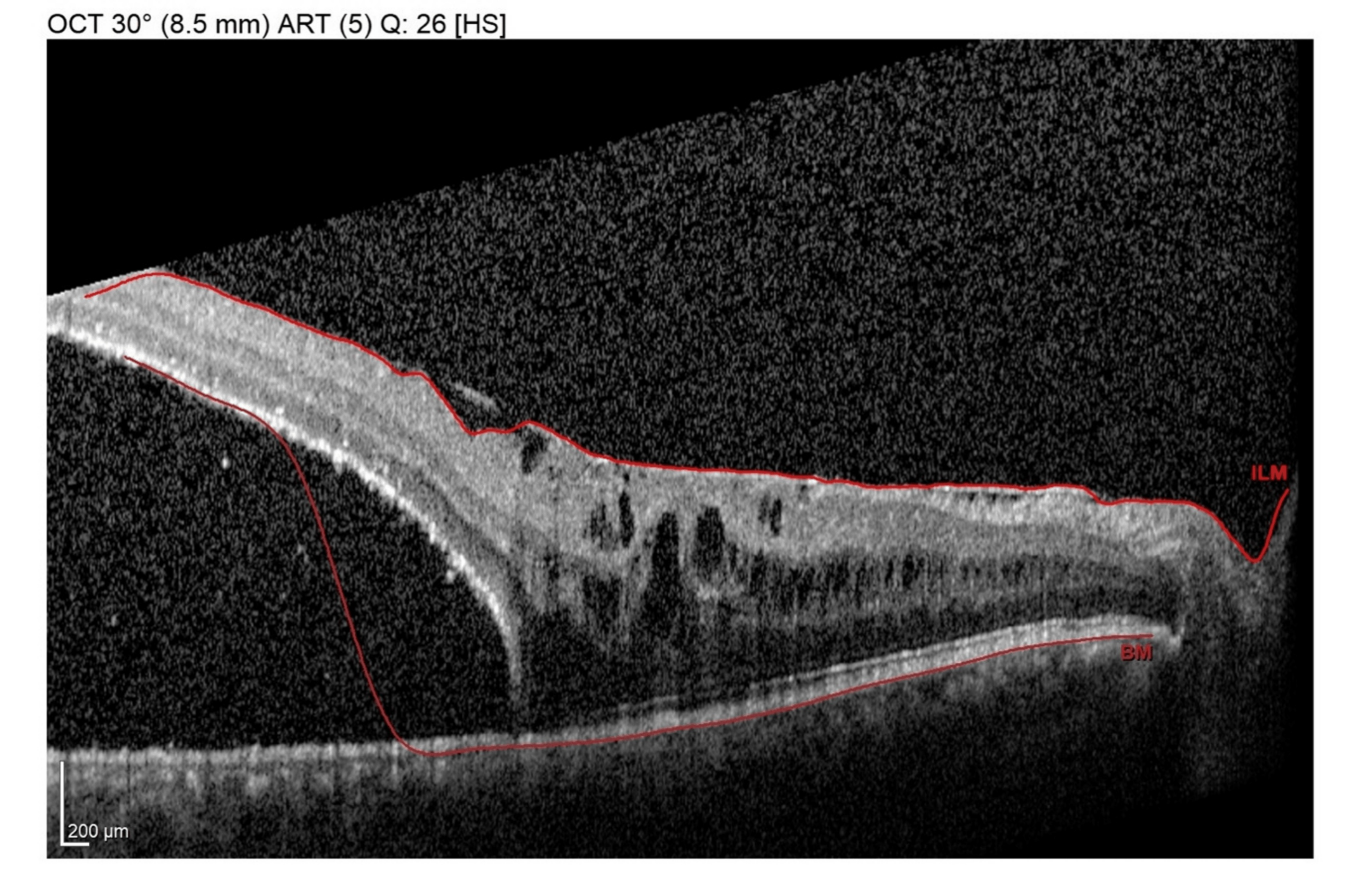
A preoperative SD-OCT line scan through the macula shows an extension of subretinal fluid involving the macula, associated with an epiretinal membrane and intraretinal cystoid changes. SD-OCT, spectral-domain optical coherence tomography

A 59-year-old, medically fit female was referred to our center with a diagnosis of right-eye RD. The patient had been experiencing flashes, floaters, and a black curtain-like vision in her right eye for the past month. She had no history of ocular trauma or prior eye interventions. Upon initial examination, her visual acuity was 20/100 in the right eye and 20/30+ in the left eye. Intraocular pressure was normal in both eyes, and the anterior segment examination was unremarkable, except for early cataract changes noted bilaterally. A thorough posterior segment, scleral-depressed examination revealed inferior temporal RD involving the macula, with inferotemporal retinoschisis extending to the inferior arcade in the right eye, while the left eye showed inferotemporal retinoschisis. No retinal breaks were identified in either eye during this examination (Figure 1). Imaging with spectral-domain optical coherence tomography (SD-OCT) confirmed the presence of subretinal fluid with RD reaching the macula in the right eye and an epiretinal membrane (Figure 2).

**Figure 3:**
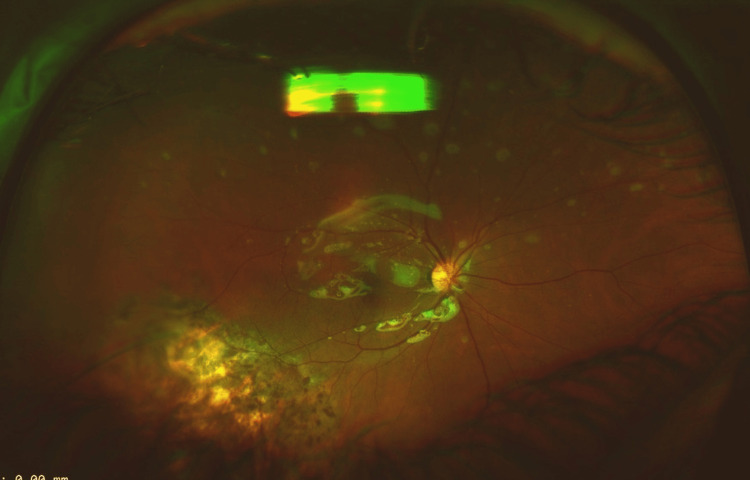
A postoperative wide-field fundus photo showing a flat retina and endolaser scars under silicone oil.

The patient underwent phacoemulsification with posterior chamber intraocular lens (IOL) implantation (+24.50 D), 25-gauge, triamcinolone-assisted pars plana vitrectomy (PPV), posterior vitreous detachment (PVD) induction, membrane peeling, internal limiting membrane (ILM) peeling, localized retinectomy, and air-fluid exchange, and silicone oil 5000 was used as tamponade. During the vitrectomy, an inferior temporal inner retinal break was localized, and two outer retinal breaks were discovered temporally, which had not been previously identified. Localized retinectomy around the inner hole was performed to overlap the inner hole with the outer breaks for effective endolaser application. Thick subretinal and intraretinal fluid were extruded by active extrusion. The patient was followed up during outpatient visits over the course of six months, during which she remained asymptomatic, with stable vision. SD-OCT scans showed a decrease in subretinal fluid and an attached retina under the silicone oil tamponade. Silicone oil was chosen as the patient lives in a high-altitude area (Figure 3).

**Figure 4:**
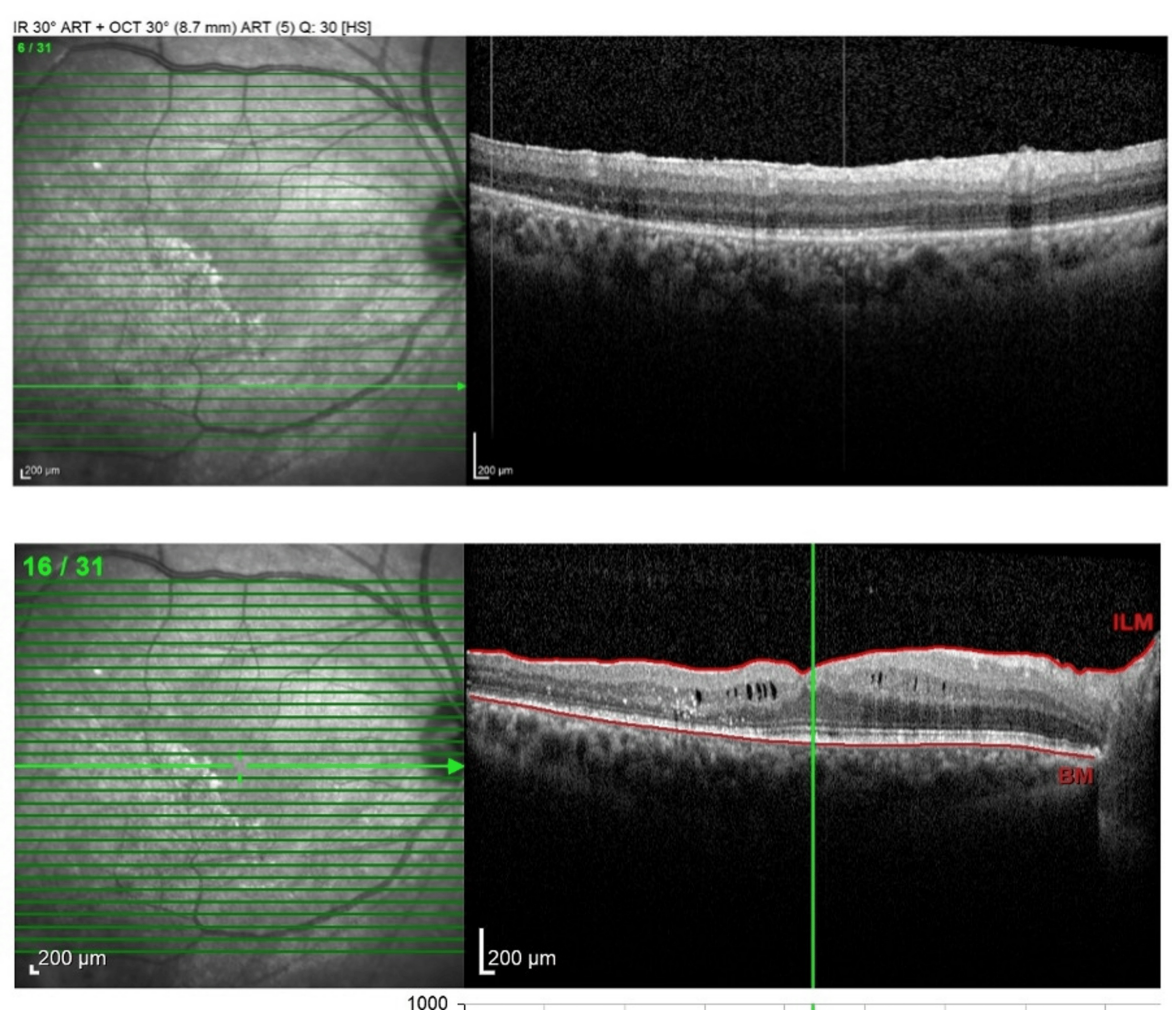
A postoperative SD-OCT horizontal scan through the inferotemporal area, 1 month after silicone oil removal, shows an attached retina (upper image) and an attached macula, with resolution of anatomical macular structures. SD-OCT, spectral-domain optical coherence tomography

At six months post-surgery, the patient underwent silicone oil removal via a 23-gauge PPV, with multiple air-fluid exchanges. After the procedure, her visual acuity improved to 20/30 in the right eye. Fundus examination of the right eye revealed a flat retina, and SD-OCT confirmed a flat macula with preserved foveal contour and no residual subretinal fluid (Figure 4). Moreover, the subjective refraction for both eyes after surgery is as follows: OD: plano -1.00 × 90, OS: plano +1.50 × 90. The patient’s postoperative course remained stable, and she was asymptomatic, with favorable visual outcomes. In this brief case report, we demonstrate successful management, with a favorable anatomical and functional outcome of a retinoschisis-related RRD.

All diagnostic modalities and clinical assessments used in this study, including fundus examination, scleral depression, and SD-OCT, represent standard clinical ophthalmic practices and do not involve proprietary classification systems, scoring tools, or licensed clinical scales. Therefore, no permissions or licensing were required for their use in this study.

## Discussion

Currently, there are no well-established therapeutic approaches to guide the best practice pattern for retinoschisis-related RRD. Various interventions have been described in the literature, including PPV, laser photocoagulation, and pneumatic retinopexy (PR) [1,3,6,7]. PPV is the most frequently performed surgery in patients with retinoschisis-associated RD. The use of scleral buckle (SB), alone or with PPV, has been studied in the literature; outcomes were significantly better in patients who underwent PPV with SB; however, no difference was reported in terms of anatomical success and the rate of reattachment [4,8,9].

Other modalities of treatment have been utilized in retinoschisis-associated RD. PR is a known method for treating RD, which involves closing the tear by laser photocoagulation and the use of intraocular gas. The use of intraocular gas in retinoschisis-associated RD has been reported in the literature; in one reported case, reattachment was required, so external drainage was used with intraocular gas injection [10,11]. Suzuki et al. reported the use of PR without external drainage in the treatment of retinoschisis-associated RD [12].

The comparison in Table 1 was carried out according to age, gender, pre- and post-operative visual acuity, macular involvement, procedure, and the tamponading agent. In our case, the patient underwent phacoemulsification with posterior chamber IOL implantation, followed by PPV, membrane peeling, and ILM peeling, and silicone oil 5000 was used as tamponade. Interestingly, the use of silicone oil as a tamponading agent was only reported once in retinoschisis-associated RD [9]. However, in Table 1, the most frequently used tamponading agent was SF6, followed by C3F8 [13,14].

**Table 1:** Summary of data for patients with retinoschisis-related retinal detachment. Studies included: [7,9,13,14] VA, visual acuity; PPV, pars plana vitrectomy; SB, scleral buckle

Ref	Demographics			Visual Acuity		Macula		Procedure			Tamponade			
	Age	M	F	Pre-op VA	Post-op VA	On	Off	PPV + SB	PPV	SB	SF6	C3F8	Silicone	Air
Garneau et al.	70	19	22	20/40	20/30	25	13	25	14	2	23	16	1	0
Beatson et al.	51	7	10	20/32	20/32	8	9	11	3	3	10	5	0	3
Gotzaridis et al.	65	17	13	NA	NA	6	24	NA	NA	17	NA	NA	NA	NA
Stem et al.	63	23	14	20/90	20/60	23	14	14	9	14	12	11	0	11

In our case, the inner retinal hole was found to be close to the outer retinal holes. Localized retinectomy, involving both inner and outer holes, was performed to facilitate drainage of the subretinal fluid and application of endolaser. Silicone oil was used, as the patient lives in a high-altitude city. The choice of surgery depends on a multitude of factors that are seldom discussed in the literature on retinoschisis-associated RRD.

## Conclusions

The current case highlighted the presentation, anatomical, and functional outcomes with treatment modalities of RS-associated RD. PPV with appropriate surgical modifications, including localized retinectomy and adequate tamponade, can result in favorable anatomical and functional outcomes. Further systematic reviews and meta-analyses of the current literature are required to compare the effectiveness and the prognosis of different treatment modalities.
